# Molecular systematics of the anchovy genus *Encrasicholina* in the Northwest Pacific

**DOI:** 10.1371/journal.pone.0181329

**Published:** 2017-07-28

**Authors:** Sébastien Lavoué, Joris A. M. Bertrand, Hui-Yu Wang, Wei-Jen Chen, Hsuan-Ching Ho, Hiroyuki Motomura, Harutaka Hata, Tetsuya Sado, Masaki Miya

**Affiliations:** 1 Institute of Oceanography, National Taiwan University, Taipei, Taiwan; 2 Department of Computational Biology, Biophore, University of Lausanne, Lausanne, Switzerland; 3 Swiss Institute of Bioinformatics, Génopode, Quartier Sorge, Lausanne, Switzerland; 4 National Museum of Marine Biology and Aquarium, Pingtung, Taiwan; 5 The Kagoshima University Museum, 1-21-30 Korimoto, Kagoshima, Japan; 6 The United Graduate School of Agricultural Sciences, Kagoshima University, 1-21-24 Korimoto, Kagoshima, Japan; 7 Department of Ecology and Environmental Sciences, Natural History Museum and Institute, Chiba, 955-2 Aoba-cho, Chuo-ku, Chiba, Japan; Tierarztliche Hochschule Hannover, GERMANY

## Abstract

The anchovy genus *Encrasicholina* is an important coastal marine resource of the tropical Indo-West Pacific (IWP) region for which insufficient comparative data are available to evaluate the effects of current exploitation levels on the sustainability of its species and populations. *Encrasicholina* currently comprises nine valid species that are morphologically very similar. Only three, *Encrasicholina punctifer*, *E*. *heteroloba*, and *E*. *pseudoheteroloba*, occur in the Northwest Pacific subregion of the northeastern part of the IWP region. These species are otherwise broadly distributed and abundant in the IWP region, making them the most important anchovy species for local fisheries. In this study, we reconstructed the phylogeny of these three species of *Encrasicholina* within the Engraulidae. We sequenced 10 complete mitochondrial genomes (using high-throughput and Sanger DNA sequencing technologies) and compared those sequences to 21 previously published mitochondrial genomes from various engraulid taxa. The phylogenetic results showed that the genus *Encrasicholina* is monophyletic, and it is the sister group to the more-diverse "New World anchovy" clade. The mitogenome-based dating results indicated that the crown group *Encrasicholina* originated about 33.7 million years ago (nearby the limit Eocene/Oligocene), and each species of *Encrasicholina* has been reproductively isolated from the others for more than 20 million years, despite their morphological similarities. In contrast, preliminary population genetic analyses across the Northwest Pacific region using four mitogenomic sequences revealed very low levels of genetic differentiation within *Encrasicholina punctifer*. These molecular results combined with recent taxonomic revisions are important for designing further studies on the population structure and phylogeography of these anchovies.

## Introduction

In the large tropical Indo-West Pacific (IWP) biogeographical region, inclusive of Hawaii Archipelago and Polynesia [1], endemic anchovies (Engraulidae; Clupeoidei) comprise about 50 species currently classified in seven genera: *Coilia*, *Encrasicholina*, *Lycothrissa*, *Papuengraulis*, *Setipinna*, *Stolephorus*, and *Thryssa* [2–5]. These seven genera do not form a monophyletic group because two of them, *Stolephorus* and *Encrasicholina*, were hypothesized to be more closely related to the "New World anchovy" clade than to other IWP genera [6–8]. New World anchovies (including *Engraulis*) along with *Stolephorus* and *Encrasicholina* make up the subfamily Engraulinae, whereas the other five IWP genera make up the subfamily Coiliinae [3,6].

The genus *Encrasicholina* currently comprises nine species inhabiting coastal waters throughout the IWP region. Six of these species have restricted geographical distributions: *E*. *purpurea* (Hawaiian Archipelago), *E*. *auster* (Fiji), *E*. *oligobranchus* (the Philippines), *E*. *macrocephala* (from the Red Sea to off the Sultanate of Oman), *E*. *gloria* (Persian Gulf and Red Sea), and *E*. *intermedia* (western Indian Ocean) [9–11]. The three other species are widely distributed from the West Indian Ocean to the Northwest Pacific: *E*. *punctifer*, *E*. *heteroloba*, and *E*. *pseudoheteroloba* [until recently, *E*. *pseudoheteroloba* was misidentified as *E*. *heteroloba*, and *E*. *heteroloba* was misidentified as *E*. *devisi*; see [12] for taxonomic revision]. These species form the major contribution of anchovy catches in many coastal fisheries in the IWP [13] including Taiwan's, where larvae of *E*. *punctifer* and *E*. *pseudoheteroloba* are targeted [14–16], and they are also important baitfish in the West Pacific [17].

Nelson [7] resurrected the genus *Encrasicholina* (first erected by Fowler [18]) for some species that were formerly classified into the genus *Stolephorus*, because these species share three derived morphological characters with the New World genera and worldwide-distributed temperate genus *Engraulis* [6]: 1) a distinctive organization of sensory canals, 2) the fusion of a tooth-plate to the first epibranchial, and 3) the fusion between the preural centrum 1 and ural centrum 1 in the caudal skeleton. Grande and Nelson [6] demonstrated that the IWP genus *Stolephorus* is the sister group of *Encrasicholina* + New World genera + *Engraulis*, based on seven morphological synapomorphies. Molecular works supported the sister group relationship between *Encrasicholina* and the clade comprising the New World genera and *Engraulis* [19–22], although each of those studies included only one or two species of *Encrasicholina* and incomplete character sampling. Therefore, previous studies have, at best, only partially addressed the monophyly of the genus *Encrasicholina*, and consequently, there is no reported morphological synapomorphy and limited genetic evidence supporting the monophyly of this genus. Species of *Encrasicholina* can be divided into two groups in regard to the profile of the head: species with a short rounded snout (*E*. *punctifer*, *E*. *gloria*, *E*. *intermedia*, and *E*. *purpurea*) and species with a longer snout (*E*. *heteroloba*, *E*. *pseudoheteroloba*, *E*. *oligobranchus*, *E*. *macrocephala*, and *E*. *auster*). In addition, rounded-snout species have a short maxilla with a blunt tip, whereas prominent-snout species have a longer maxilla with a pointed tip [3,12].

Species of *Encrasicholina* are small (7–10 cm in max. size) and are almost exclusively found within the coastal zone. The notable exception is *E*. *punctifer*, a species which prefers both neritic and oceanic waters [23]. Species of *Encrasicholina* for which biological data exist (i.e., *E*. *punctifer*, *E*. *pseudoheteroloba* [= *E*. *heteroloba* or *Stolephorus heteroloba* in earlier publications], *E*. *heteroloba* [= *E*. *devisi* or *Stolephorus devisi* in earlier publications], and *E*. *purpurea*), exhibit broad similarities in characteristics as they grow rapidly, attain sexual maturity in only a few months, and have a short lifespan of less than 1 year [14,24,25]. These species are multiple spawners over extended periods (sometimes extending throughout the year), but the interannual variability in recruitment is often high [26].

The fossil record of anchovies is considered disproportionately poor given their high abundance, with only a few fossil species known from the Neogene [6]. This observation is further corroborated by two recent molecule-based dating studies showing that the family Engraulidae may be as old as the late Cretaceous (i.e., 70–90 million years old) [19,22]. In 2016, however, a new and exceptional (given the rarity of anchovies in the fossil record) fossil specimen was described from the locality Monte Bolca (in northern Italy) as a new genus and a new species of anchovy, †*Eoengraulis fasoloi* [27]. This fossil somewhat fills the gap between the molecular time estimation and the fossil record information. Marramà and Carnevale [27] placed this fossil as the sister group of the subfamily Engraulinae, therefore, providing a strict minimum age for the crown group Engraulidae to about 50 million years ago (Ma). There is no fossil of *Encrasicholina*.

Herein, we studied the molecular systematics of the three most-widely distributed species of *Encrasicholina* (*E*. *heteroloba*, *E*. *pseudoheteroloba*, and *E*. *punctifer*) that occur in the Northwest Pacific region by sequencing five complete mitogenomic sequences using high-throughput DNA sequencing technology. To broaden the taxonomic comparison of available mitogenomic data, we also sequenced the complete mitogenomes of five additional anchovy species that occur in the Northwest Pacific region (*Engraulis japonicus*, *Setipinna tenuifilis*, and *Thryssa dussumieri*) and elsewhere (*Thryssa setirostris* and *Anchoviella jamesi*), using both high-throughput and Sanger sequencing technologies. Finally, our dataset offered a first (although limited) insight on the genetic differentiation within *Encrasicholina punctifer*.

## Materials and methods

### Ethics statement

This research was performed at the Natural History Museum & Institute (Chiba, Japan) and National Taiwan University (Taipei, Taiwan) in accordance with these institutions' guidelines regarding animal research. No ethics statement was required for this project as no experiment involved live fishes, and none of the species examined in this study is listed on the checklist of CITES (http://checklist.cites.org) or is under local protection policies. Seven fresh specimens examined in this study were purchased from local fish markets in Taiwan (4 specimens, Taiwan Strait, Anping fish market nearby Tainan City and Dong-shi fish market in Chiayi), Japan (2 specimens; Uchinoura Bay, Kagoshima fish market), and Thailand (one specimen, Andaman Sea, Phuket fish market); one specimen of *E*. *punctifer* (KAUM—I. 60438) was collected during a research cruise of R/V Kumamoto-maru in the East China Sea at 28°16.14'N, 123°14.52'E (in international waters); the specimen of *Anchoviella jamesi* was obtained from an ornamental fish supplier in Japan, "Aquashop Ishi to Izumi" (http://www.ishitoizumi.com/), and we euthanized it with an overdose of the anesthetic MS-222. The tissue samples from the Philippines were taken under a Memorandum of Agreement for joint research made by and among the Department of Agriculture of the Republic of the Philippines (DA), the University of the Philippines Visayas (UPV), the Kagoshima University Museum, the Research Institute for Humanity and Nature, and Tokai University, facilitated by S. L. Sanchez [Bureau of Fisheries and Aquatic Resources (BFAR), DA]. P. J. Alcala (DA) provided a Prior Informed Consent Certificate, and I. P. Cabacaba and S. M. S. Nolasco (BFAR, DA) provided a fish sample Export Certificate (No. 2016–39812). A tissue sample of a specimen of *Thryssa setirostris* was obtained through a legal tissue donation from the Universiti Sains Malaysia, an international research institute.

### Sample preservation and taxonomic sampling

A small piece of muscle or fin was taken from each specimen and immediately fixed in 95% ethanol. The whole body was preserved in formaldehyde or ethanol. Combining our mitogenome sequences with sequences archived in GenBank, the taxonomic sampling included a total of 32 specimens representing 25 species of anchovies (Table 1). In all analyses, the family Engraulidae was assumed to be monophyletic, and *Ilisha elongata* (Pristigasteridae; Clupeoidei) was used to root the trees. Therefore, the root corresponded to crown-group Clupeoidei, because Engraulidae was hypothesized to be the sister group of the rest of the Clupeoidei, including the Pristigasteridae [4].

**Table 1:** List of taxa examined in this study with familial and subfamilial classifications indicated. Within *Encrasicholina*, the nomenclature follows the recent revision by Hata and Motomura [9]; see text for details.

Classification	Species	Origin	Accession Nos.	Reference
Order Clupeoidei				
Family Pristigasteridae	*Ilisha elongata*	Northwest Pacific, Japan	AP009141	Lavoué et al. [59]
Family Engraulidae				
Engraulinae	*Engraulis japonicus*	Northwest Pacific, Japan	AB040676	Inoue et al. [60]
	*Engraulis japonicus* (S18)	Northwest Pacific, Taiwan	**AP017957**	**This study**
	*Engraulis encrasicolus*	East Atlantic, France	AP009137	Lavoué et al. [20]
	*Anchoviella cf guianensis* (LBP 2297)	New World, South America	AP011557	Lavoué et al. [20]
	*Anchoviella cf jamesi* (CBM-ZF-12586)	New World, South America	**AP012524**	**This study**
	*Lycengraulis grossidens*	New World, South America	AP011563	Lavoué et al. [20]
	*Amazonsprattus scintilla*	New World, South America	AP009617	Lavoué et al. [20]
	*Encrasicholina punctifer*	Mariana Trench, West Pacific	AP011561	Lavoué et al. [20]
	*Encrasicholina punctifer* (S17)	Northwest Pacific, Taiwan	**AP017955**	**This study**
	*Encrasicholina punctifer* (KAUM-I 60438; S12)	East China Sea	**AP017951**	**This study**
	*Encrasicholina punctifer* (CBM-ZF 14731; S15)	Northwest Pacific, Japan	**AP017956**	**This study**
	*Encrasicholina heteroloba* (KAUM-I 56997; S14)	Northwest Pacific, Japan	**AP017952**	**This study**
	*Encrasicholina pseudoheteroloba* (KAUM-I 59682; S21)	Andaman Sea, Thailand	**AP017954**	**This study**
	*Stolephorus* sp AP011566	Southeast Asia, Thailand	AP011566	Lavoué et al. [20]
	*Stolephorus* sp AP011567	Central Indian Ocean	AP011567	Lavoué et al. [20]
	*Stolephorus commersonii*	West Pacific, China	KX753639	Retrieved from Genbank
Coiliinae	*Thryssa baelama*	Southeast Asia	AP009616	Lavoué et al. [20]
	*Thryssa setirostris* (Universiti Sains Malaysia; S24)	Southeast Asia, Malaysia	**AP017958**	**This study**
	*Thryssa dussumieri* (NTUM-12724; S09)	Northwest Pacific, Taiwan	**AP017953**	**This study**
	*Thryssa kammalensis*^a^	Northwest Pacific, China	KT985048	Zhang and Gao [61]
	*Setipinna melanochir*	Mekong R., Cambodia	AP011565	Lavoué et al. [20]
	*Setipinna tenuifilis* (NMMB-P23498; S10)	Northwest Pacific, Taiwan	**AP017950**	**This study**
	*Lycothrissa crocodilus*	Southeast Asia, Cambodia	AP011562	Lavoué et al. [20]
	*Coilia lindmani*	Southeast Asia, Cambodia	AP011558	Lavoué et al. [20]
	*Coilia reynaldi*	central Indian Ocean	AP011559	Lavoué et al. [20]
	*Coilia brachygnathus*	Northwest Pacific, China	KP185129	Wang et al [62]
	*Coilia ectenes*	Northwest Pacific, China	JX625133	Qiao et al [63]
	*Coilia nasus*	Northwest Pacific, China	KM276661	Zhao et al [64]
	*Coilia nasus*	Northwest Pacific, Japan	AP009135	Lavoué et al. [59]
	*Coilia grayii*	Northwest Pacific, China	KF938994	Li et al [65]
	*Coilia mystus*	Northwest Pacific, China	KF056322	Retrieved from Genbank
	*Coilia mystus*	Northwest Pacific, China	JX534238	Zhang et al [66]

^a^ Likely *Thryssa chefuensis* (see [3,67]).

### DNA extraction and mitochondrial genome sequencing

First, we extracted genomic DNA from the tissue samples using a commercial kit (DNeasy Blood and Tissue Kit, Qiagen, Hilden, Germany), following manufacture's protocol. All ten mitogenomes were amplified with a long Polymerase Chain Reaction (PCR) amplification technique into four overlapping fragments [28], following the standard laboratory protocol described in Miya and Nishida [29]. The primer sequences to amplify the four long fragments are: L12321-leu (5'- GGT CTT AGG AAC CAA AAA CTC TTG GTG CAA-3'), L2508-16S (5'-CTC GGC AAA CAT AAG CCT CGC CTG TTT ACC AAA AAC-3'), L8343-lys (5'- AGC GTT GGC CTT TTA AGC TAA WGA TWG GTG-3'), H12293-leu (5'-TTG CAC CAA GAG TTT TTG GTT CCT AAG ACC-3'), H1065-12S (5'- GGC ATA GTG GGG TAT CTA ATC CCA GTT TGT-3'), H15149-cytb (5'- GGT GGC KCC TCA GAA GGA CAT TTG KCC TCA-3') and HS-LA-16S (5'- TGC ACC ATT RGG ATG TCC TGA TCC AAC ATC-3'). For nine specimens (see Table 1), the long PCR products were sequenced using high-throughput DNA sequencing technology as following: the genetic libraries were prepared from the long PCR products using Nextera XT DNA Library Preparation Kit (Illumina, San Diego, USA) following manufacture's protocol and then sequenced using a MiSeq Sequencing platform (Illumina) at Natural History Museum and Institute, Chiba. The long PCR products of *Anchoviella jamesi* were used as templates to amplify short (<1500 bp), contiguous and overlapping segments of the mitogenome using short PCR technique [29]. Short PCR fragments were purified using an ExoSap enzyme reaction, before being used as templates for direct cycle sequencing with dye-labeled terminators (Sanger sequencing technology). All sequencing reactions were performed according to the manufacturer’s instructions (Applied Biosystems, Foster City, USA) with sequencing primers used as the same as those used for PCR. Labeled fragments were run on a 3130*xl* Genetic Analyzer (Applied Biosystems).

### Mitogenome reconstruction and annotation

To reconstruct the fish mitochondrial genome sequence of each individual from read data generated by a high-throughput sequencing technique, we used the baiting and iterative mapping procedure implemented in MITObim v1.8 [30]. Raw reads were first trimmed by quality with the *FASTQ Quality Trimmer* script [31] available in the online Galaxy portal (www.usegalaxy.org). Reads were trimmed at both the 5' and 3' ends until the aggregate quality score was ≥ 20 (all other settings were kept to default values). We performed reconstructions following two main approaches available in the MITObim pipeline. We first used as a starting reference previously published mitochondrial genomes of taxa that are closely related to the target species (Table 2). We then used conspecific (or congeneric) COI sequences as a seed to initiate the process. The program was used with the option—pair, and baiting stringency was lowered (*—kbait* < 31) for some individuals for which the process could not be initiated (all others settings were kept to default values). The circularity of the mitochondrial genomes was inferred thanks to editing features provided in Geneious 6.1.8 [32], and raw reads were mapped-back onto the result sequences to check for assembly success and assess coverage.

**Table 2:** Characteristics of high-throughput sequencing data (Illumina) for each reconstructed mitogenome and corresponding reference mitochondrial genome information.

Species	Individual ID	Inferred Size (bp)	% GC content	Mean Coverage	SD Coverage	Minimum Coverage	Maximum Coverage	Number of reads	Accession reference *mt* genome
*Encrasicholina punctifer* (KAUM—I. 60438)	S12	16,682	45.5	1247.8	1307.7	2	4406	114,632	**KF765500** (*Engraulis japonicus*)
*Encrasicholina punctifer* (CBM-ZF 14731)	S15	16,695	45.5	2816.4	2105.4	62	7826	230,072	**KF765500** (*Engraulis japonicus*)
*Encrasicholina punctifer* (S17)	S17	16,682	45.5	869.7	757.8	16	2442	79,318	**KF765500** (*Engraulis japonicus*)
*Engraulis japonicus* (S18)	S18	16,675	46	1697.9	1255.2	143	4650	145,594	**KF765500** (*Engraulis japonicus*)
*Encrasicholina pseudoheteroloba* (KAUM—I. 59682)	S21	16,670	43.7	1654.4	1567.8	14	4930	137,162	**KF765500** (*Engraulis japonicus*)
*Encrasicholina heteroloba* (KAUM—I. 56997)	S14	16,665	44.9	1397.3	959	224	3898	143,748	**KF765500** (*Engraulis japonicus*)
*Thryssa setirostris* (Malaysia)	S24	16,879	46.1	928.3	1043.8	9	3335	72,558	**AP009616** (*Thryssa baelama*)
*Thryssa dussumieri* (NTUM-12724)	S9	16,920	44.5	1422.1	1803.3	3	12596	139,258	**AP009616** (*Thryssa baelama*)
*Setipinna tenuifilis* (NMMB-P23498)	S10	16,884	44.7	1669.1	2252.5	16	18111	168,336	**KC439458** (*Setipinna taty*)

Specimens from Taiwan deposited at National Taiwan University Museums (NTUM) and National Museum of Marine Biology and Aquarium, Pingtung (NMMB-P).

For *Anchoviella jamesi*, the sequence electropherograms were edited with EditView version 1.0.1 (Applied Biosystems). Sequencher software package version 4.1.2 (Gene Codes, Ann Arbor, MI, USA) and DNASIS version 3.2 (Hitachi Software Engineering, Yokohama, Japan) were used to concatenate the consensus mitogenomic sequence.

The consensus sequences were annotated using the pipeline "MitoAnnotator" of MitoFish [33] and then exported for analyses. The gene content and order of the newly determined mitogenomic sequences were typical of those found in most other teleosts [34]. Table 1 provides information on the specimens included in our study, including accession numbers for mitogenome data archived in the DDBJ/EMBL/GenBank database.

Across the 33 sequences considered herein (i.e., 32 ingroup taxa plus one outgroup), sequences at each protein-coding gene were manually aligned with respect to the translated amino acid sequence except for the ND6 gene that was excluded from subsequent phylogenetic analyses because of its heterogeneous base composition. The 12S and 16S ribosomal RNA (rRNA) sequences, as well as the concatenated 22 transfer RNA (tRNA) genes, were aligned with the software Proalign vers. 0.5 [35] using default parameter settings. Regions with posterior probabilities of ≤ 90% were excluded from subsequent analyses. The aligned data matrix (14,625 positions in total) included concatenated nucleotide sequences from 22 tRNA genes (1567 positions) and the two rRNA genes (2183 positions) plus the codon positions of 12 protein-coding genes (10,875 positions). The pairwise uncorrected genetic distances between mitogenomes of *Encrasicholina punctifer* were calculated using the software Sequencher on the total length of the mitogenomes.

### Phylogenetic analyses and divergence time estimation

We first inferred partitioned maximum likelihood (ML) phylogenetic trees using the software RAxML [36] with its graphical interface, raxmlGUI 0.9Beta3 [37] from the mitogenomic matrix previously built. We used PartitionFinder v1.1.1 [38] to calculate the best partition scheme from 38 basic partitions (i.e. the first, second and third positions of each coding-protein nuclear genes along with the concatenated 12S/16S rRNAs and the concatenated 22 tRNA genes). A 21 partitions scheme was inferred and for each of these partitions, we applied a general time-reversible model of sequence evolution with gamma rate variation ("GTR + G" model) and four discrete rate categories.

We performed ML heuristic phylogenetic searches under the general time reversible model with discrete gamma-distributed rate heterogeneity [GTR + G] and data partitioning as described above. We performed 100 searches for each three analyses and found the best ML tree by comparing final likelihoods among the 100 inferred trees. To evaluate the robustness of the internal branches of the ML tree, 1000 bootstrap replicates were calculated for each matrix under the GTR + G model.

We then simultaneously inferred the phylogeny and divergence times (with their 95% credibility intervals) using a partitioned Bayesian method that incorporated a relaxed molecular clock, as implemented in MrBayes v3.2.2 [39]. The matrix was partitioned as before, and the GTR + G model of sequence evolution was again chosen for each of the 21 data partitions, with parameters unlinked between partitions. The relaxed molecular clock followed a lognormal prior with an uncorrelated independent gamma rates (IGR) model.

The age of the Engraulidae and the age of the tree root were constrained using the latest paleontological information as indicated hereafter. We enforced the monophyly of the taxon "Engraulidae" in order to root the tree, thereby constraining *Ilisha elongata* to be the outgroup. Each of the two age-constraints followed an exponential distribution with a strict minimum age and a relaxed maximum age within the 95% credibility interval (95% CI). Two independent MCMC runs were initiated in parallel for 50 million generations, sampling the trees every 5,000 generations with the first 25% of samples discarded as burn-in and the remaining tree samples, from the two runs, pooled together. Each run’s parameters were checked for convergence with the Tracer v.1.6 software [40]. The maximum clade credibility tree with mean divergence times and 95% CIs were automatically calculated from the combined tree samples in MrBayes.

Lavoué et al [19] discussed the quality of the fossil record of Clupeoidei. Hereafter, we update their discussion because several paleontological works published after 2013 significantly improved the knowledge on the evolution of Clupeoidei with the descriptions of several new taxa and the taxonomic revisions of some others. There are, however, three critical issues that still limit the use of many clupeoid fossils in molecular dating: 1) the families Dussumieriidae and Clupeidae are likely reciprocally not monophyletic [19,22,41]; 2) the higher taxonomic-level phylogeny of Clupeoidei is still mostly unresolved [4]; and 3) older clupeoid fossils often exhibit puzzling combinations of morphological characters relative to extant taxa [42–44]. Altogether, this makes difficult to elucidate the phylogenetic positions of several clupeoid fossils.

The Clupeoidei appeared in the fossil record of the Late Cretaceous with two freshwater taxa from South America, †*Pseudoellimma gallae* (Barremian; 129.4–125.0 Ma) [45] and †*Cynoclupea nelsoni* (family †Cynoclupeidae; limit Barremian/Aptian; 125.0 Ma) [43]. While †*Pseudoellimma gallae* is considered a stem clupeoid [45], Malabarba and Di Dario [43] suggested that †*Cynoclupea nelsoni* is a crown clupeoid. Therefore, using †*Cynoclupea nelsoni*, we constrained the minimum age of the root of our tree (which corresponds to crown group Clupeoidei) to 125 Ma and relaxed the 95% CI maximum age to 145 Ma (limit Jurassic/Cretaceous) because of the absence of any Jurassic clupeoid, clupeiform and clupeomorph fossils. This interval of time (145–125 Ma) is reasonably congruent with the overall fossil record of the Clupeoidei [42–51] and more generally, with the fossil record of the Teleostei [52].

Recent paleontological works showed that during the Late Cretaceous and the early Cenozoic, the clupeoids greatly diversified in forms and space. Fossils include †*Garganoclupea svetovidovi* (†Garganoclupeidae) and †*Apricenaclupea ridewoodi* (Clupeidae) from the Santonian (Italy, Apricena) [51], †*Nolfia riachuelensis* (Clupeidae) from the Albian (Brazil) [42], †*Lecceclupea ehiravaensis* and †*Italoclupea nolfi* (Clupeidae) from the Campano-Maastrichthian (Italy, Nardò) [47,48], †*Trollichthys bolcensis* (Dussumieriidae) [44], †*Bolcaichthys catopygopterus* (Clupeidae) [49] and most importantly for this work, †*Eoengraulis fasoloi* (Engraulidae) from the Eocene (Italy, Monte Bolca) [27]. Whereas the phylogenetic positions of most of these fossils are unresolved, Marramà and Carnevale [27] strongly suggested that †*Eoengraulis fasoloi*, is the sister group of the subfamily Engraulinae. Consequently, this fossil provides a strict minimum age of 50 Ma for the most recent common ancestor of the Engraulidae. The 95% CI maximum age was set to 86.3 Ma corresponding to the limit Coniacian/Santonian because most of the crown group clupeoid fossils are younger.

## Results and discussion

### High-throughput mitogenomic sequence quality and assembly

For each specimen, reads corresponding to mitochondrial genome were effectively identified from total sequence reads with a sample-specific indexing system. After sequence trimming by removing low-quality sequences, the read number per specimen varied from 72,558 to 230,072 (see Table 2 for details).

From these data, we successfully reconstructed complete circular mitogenomes for all of the specimens (see Table 2 for details). Both methods of consensus reconstruction, as implemented in MITObim, provided highly concordant results through the entire sequence even if we noted some discordances in a few very limited fragments (the length of which was always < 1% of the total mitogenome sequence). These discordances were often associated with regions containing repeated elements where uncertainty in read mapping could have altered the reconstruction process. Therefore, we decided to remove these potentially problematic blocks from the final alignments. Despite differences in read coverage that might be attributed to unequal concentrations of polymerase chain reaction (PCR) products in the mix, the overall length and the good quality of the paired-end reads allowed us to check the reliability of the consensus sequences inferred (with a mean coverage > 869 X).

### Phylogenetic and dating results

**Fig 1:**
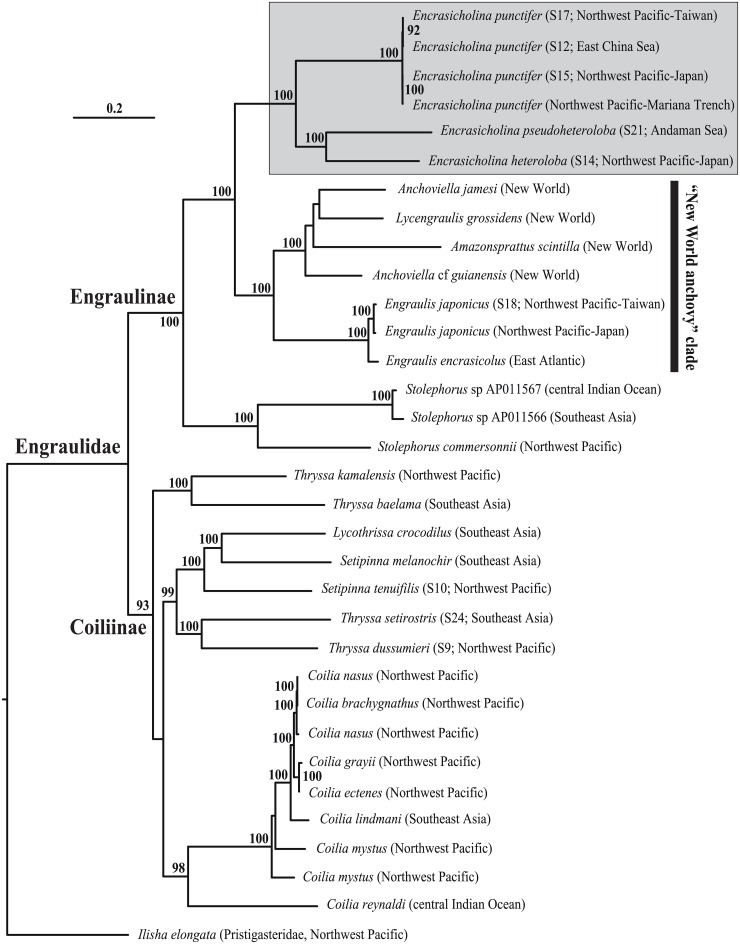
Maximum likelihood tree of the Engraulidae from analysis of the mitogenomic matrix. Branch lengths are proportional to the number of substitutions per nucleotide position (scale bar = 0.3 substitutions). Numbers at nodes are Bootstrap Proportions (indicated in percentage). The tree is rooted with *Ilisha elongata* (Pristigasteridae). The genus *Encrasicholina* is highlighted in grey. See text for details on the method of phylogenetic reconstruction.

The ML analysis yielded a fully resolved phylogenetic tree with most of the relationships strongly supported by high bootstrap proportions (BPs) (Fig 1). In this tree, the family Engraulidae was divided into two clades corresponding to the two subfamilies Coiliinae and Engraulinae (BPs = 100%); this is congruent with the results of several morphological and molecular studies [6,8,19,20,22]. The Coiliinae comprises the sampled IWP genera *Coilia*, *Thryssa*, *Lycothrissa*, and *Setipinna*, whereas the Engraulinae includes the IWP genera *Stolephorus* and *Encrasicholina* along with the "New World anchovy" clade representative genera *Lycengraulis*, *Amazonsprattus*, *Anchoviella*, and *Engraulis*.

The genus *Encrasicholina* formed a monophyletic group (BP = 100%) with *E*. *punctifer* being the sister group of *E*. *pseudoheteroloba* plus *E*. *heteroloba* (BP = 100%). *Encrasicholina* was the sister group of the "New World anchovy" clade (BP = 100%). Despite high morphological similarities among species of *Encrasicholina*, in particular between *E*. *pseudoheteroloba* and *E*. *heteroloba*, each of the three species lineages was genetically well distinct; this indicates that genetic and morphological differentiations are decoupled in *Encrasicholina* when compared to other anchovy groups [20,21].

In addition to these phylogenetic results and although our taxonomic sampling was still far from comprehensive within the subfamily Coiliinae, we detected a strong signal to support: 1) the paraphyly of the genus *Setipinna* relative to *Lycothrissa* [5]; and 2) the polyphyly of the genus *Thryssa* which comprises two independent lineages. The first lineage comprises *Thryssa dussumieri* and *Thryssa setirostris*, two species with long maxilla, whereas the second lineage includes *Thryssa baelama* and *Thryssa kammalensis*, two species with much-shorter maxilla [3].

The taxonomy and nomenclature of the genus *Thryssa* are complicated, and they are in need of a thorough revision. The non-monophyly of this genus, as found herein, adds difficulties to these problematic taxa. Whereas Grande and Nelson [6] recognized the genus *Thrissina* for *Thrissina baelama*, Whitehead et al. [3] synonymized it with *Thryssa*. According to Whitehead et al. [3], *Thryssa* comprises 24 species classified in three subgenera: *Thryssa* (type species: *T*. *setirostris*), *Thrissina* (type species: *T*. *baelama*), and *Scutengraulis* (type species: *T*. *hamiltonii*). Kottelat [53] pointed out that *Thryssa* is not a valid name, and he proposed replacing it with *Thrissina*. Eschmeyer et al. [2], however, did not follow Kottelat [53] and retained the name *Thryssa* for the sake of stability. If the genus *Thryssa* sensu [3] is confirmed not to be monophyletic, with *T*. *setirostris* and *T*. *baelama* belonging to two independent lineages, two generic names will be necessary for these lineages. Before introducing any taxonomic or nomenclatural changes, the study of a denser taxonomic sampling within *Thryssa* is necessary to better identify the content of each lineage.

**Fig 2:**
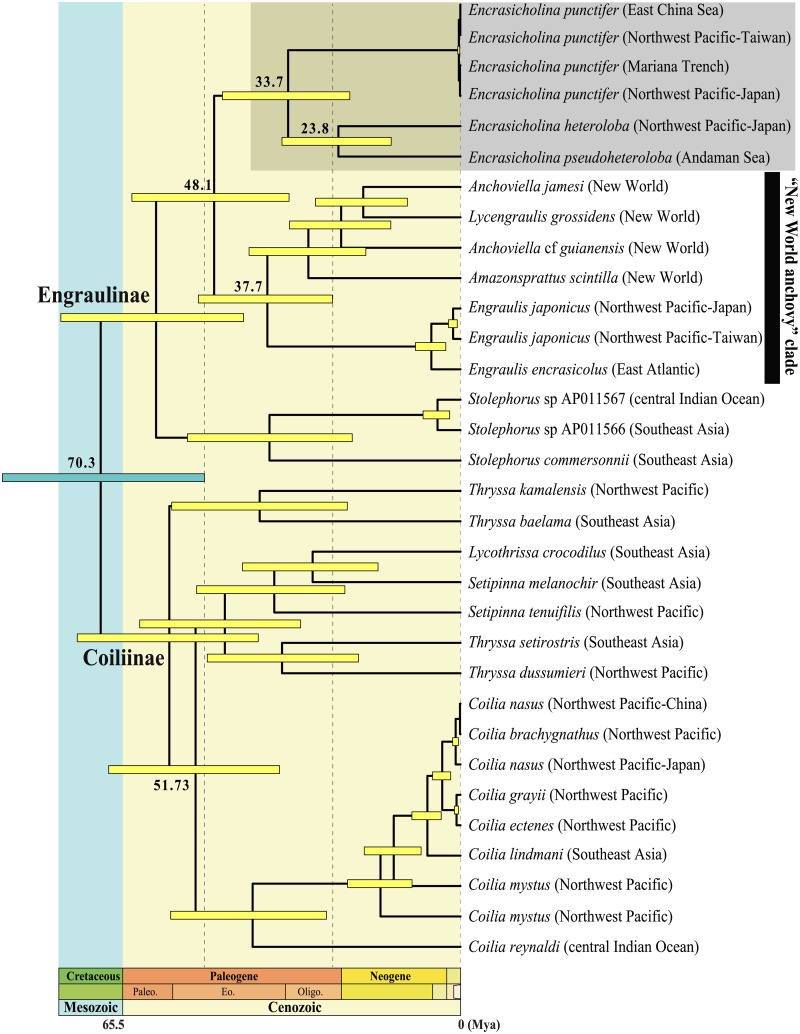
Phylogenetic chronogram of the Engraulidae based on a Bayesian relaxed clock analysis. The outgroup *Ilisha elongata* is not shown. Horizontal timescale is in million years before present (Ma) (Paleogene Epoch abbreviations: Paleo, Paleocene; Eo, Eocene; and Oligo, Oligocene). The yellow and grey horizontal bars at nodes are 95% age credibility intervals. The grey horizontal bar indicates calibration constraint of Engraulidae age. Numbers in italics given at nodes are the Bayesian posterior probabilities when <1. See text for details on the method of time-calibrated phylogenetic reconstruction.

The topology of the Bayesian timetree (Fig 2) was the same as the topology of the ML phylogenetic tree inferred from the same matrix and data partitioning. Using the age of †*Eoengraulis fasoloi* to constrain the minimum age of the crown group Engraulidae and setting the divergence Pristigasteridae/Engraulidae within the range of 145–125 Ma, we inferred the age of the most recent common ancestor of Engraulidae (i.e., the age of the crown group) to 70.3 Ma [95% CI = 89.5~50.1 Ma].

Three recent time-calibrated phylogenetic trees have been published for the family Engraulidae [19,22,54]. The age estimation of the family Engraulidae among these three studies varied by a factor of almost 10, from only 9.3 Ma (95% CI = 10.2~8.5 Ma) in [54] to about 89 Ma (95% CI = 100~80 Ma) in [22]. The estimation of Silva et al [54] is in conflict with the fossil record. For example, the oldest crown group engraulid, †*Eoengraulis fasoloi*, is 40 My older than the age of the crown group Engraulidae inferred in Silva et al [54]. Similarly, †*Cynoclupea nelsoni* provides a strict minimum age of 125 Ma for the divergence between *Denticeps* (Denticipitoidei) and the Clupeoidei whereas Silva et al [54] estimated this divergence to only 22 Ma.

Bloom and Lovejoy [22] estimated the age of the Engraulidae to about 89 Ma (95% CI = 100~80 Ma), this is almost 20 My older than our estimation. We point out three potential caveats regarding to the fossil selection and the phylogeny results in [22] which could explain the difference with our estimation: 1) Bloom and Lovejoy [22] used the Late Cretaceous-Paleocene †*Gasteroclupea branisai*, which was considered a stem pristigasterid at that time, to constrain the time divergence between Pristigasteridae and Engraulidae. However, Marramà and Carnevale [55] showed that this fossil is not a pristigasterid and even not a clupeiform. According to Marramà and Carnevale [55], †*Gasteroclupea branisai* belongs to the sister group of Clupeiformes, the †Ellimmichthyiformes; 2) Bloom and Lovejoy [22] used the oldest clupeid, †*Nolfia riachuelensis*, to calibrate the age of Clupeidae (including *Sundasalanx*). According to De Figueiredo [42], however, the phylogenetic position of †*Nolfia riachuelensis* within the Clupeidae is rather uncertain and, furthermore, the family Clupeidae is not monophyletic relative to the family Dussumieriidae and the relationships among the main clupeoid lineages are still not resolved; 3) Bloom and Lovejoy [22] recovered *Denticeps* (Denticipitoidei) as the sister group of the rest of the Otocephala and not of the Clupeoidei as it is supported by morphological data and by most of the recent molecular studies [50,56,57]. Bloom and Lovejoy [22] used the oldest crown group Otocephala (= Ostarioclupeomorpha), †*Tischlingerichthys viohli* (Thitonian; 149 Ma) [58], to calibrate the divergence between Ostariophysi and the Clupeoidei, excluding *Denticeps*. Therefore, their estimation necessarily overestimated the age of Otocephala and, consequently, the age of Engraulidae.

Using a different and non-overlapping set of fossils along with a different taxonomic sampling, we note that the overall time divergence of Engraulidae in Lavoué et al. [19] is rather congruent with our estimation.

Fig 2 shows that the crown group *Encrasicholina* originated about 33.7 Ma (nearby the limit Eocene/Oligocene) [95% CI = 46.5~21.6 Ma], and each of the three species lineages of *Encrasicholina* was already separated 23.8 Ma (nearby the limit Oligocene/Miocene) [95% CI = 34.8~13.5 Ma]. It is noteworthy to mention that *Encrasicholina* and its sister group the "New World anchovy" clade began to diversify at about the same period (Oligocene), but they then experienced diametrical opposite evolutionary trajectories. *Encrasicholina* comprises only nine species that are morphologically and ecologically very similar, and they all occur in the IWP region, whereas the "New World anchovy" clade, beside the fact it occurs in a different region, is by far more speciose (about ten times more so), more diverse morphologically (e.g., paedomorphic *Amazonsprattus* or sabertooth *Lycengraulis*), and more diverse ecologically (e.g., marine and freshwater species). In the context of the phylogeny of the Engraulinae, *Encrasicholina* appears to have retained several ancestral characters, whereas conditions observed in the "New World anchovy" clade are more derived and diversified.

### Biogeography insights

Anchovies are widely distributed in the world, with most species living in marine tropical environments, and few species secondarily adapted to marine temperate environments and freshwater tropical environments [19,21]. Anchovies likely originated in the proto-IWP region when this region was connected to the Atlantic Ocean through the Tethys Sea [19]. This scenario is also indirectly supported by the oldest anchovy excavated, †*Eoengraulis fasoloi*, which lived during the Eocene in the Tethys region (currently northern Italy) [27].

Our study provides further insights into the historical biogeography of these fishes and their interoceanic distribution. It shows that the most recent common ancestor of the clade comprising the "New World anchovy" clade and *Encrasicholina* lived about 48 Ma, well before the closure of the Tethys Sea that is dated to about 23 Ma, when the Afro-Arabic plate collided with the Eurasian plate. The closure of the Tethys Sea is considered to have had important consequences for the biogeography of marine organisms. However, within our time-calibrated phylogenetic framework, the hypothesis that it was the cause of the divergence between the "New World clade" and *Encrasicholina* was rejected.

### Intraspecific differentiation in *Encrasicholina punctifer*

The three complete mitogenomes determined in this study for *Encrasicholina punctifer* (from Taiwan, the East China Sea, and Japan) were very similar to each other. They are also very similar to a previously determined partial mitogenome (about 12,000 bp) of a specimen collected near the Mariana Trench [20]. There are a maximum of 42 substitutions between specimens S15 and S17 (pairwise genetic distance ~ 0.25%) and a minimum of six substitutions between specimens S17 and S12 (pairwise genetic distance ~ 0.04%). In particular, we detected only one substitution in the COI gene and no substitution in the cytochrome *b* gene among the four specimens examined. These two genes are often used in population genetic analyses because of their fast rate of evolution. The small genetic divergence found here tends to indicate that the population of *E*. *punctifer* in this region is of recent origin with extremely low genetic differentiation or that substantial amount of intraspecific gene flow has occurred among populations of *E*. *punctifer* at a broad spatial scale. These preliminary results should be useful when choosing appropriate genetic markers to further examine the population genetics of this species.
